# Comparative study on coating CdSe nanocrystals with surfactants

**DOI:** 10.1007/s00604-013-1062-z

**Published:** 2013-08-08

**Authors:** Sławomir Oszwałdowski, Kenneth P. Roberts

**Affiliations:** 1 Faculty of Chemistry, Department of Analytical Chemistry, Warsaw University of Technology, ul. Noakowskiego 3, 00-664 Warsaw, Poland; 2 Department of Chemistry and Biochemistry, The University of Tulsa, Tulsa, OK 74104 USA

**Keywords:** Semiconductor nanocrystals, Surfactants, Dispersion, Optical spectra, Cloud-point extraction

## Abstract

We have synthesized CdSe nanocrystals (NCs) in sizes from 2.2 to 5.1 nm passivated with hydrophobic trioctylphosphine oxide (TOPO) in combination trioctylphosphine (TOP) or tributylphosphine (TBP) to obtain particles of the type CdSe/TOPO/TOP or CdSe/TOPO/TBP. These NCs were then dispersed in aqueous solution of ionic or non-ionic surfactants (such as stearate, oleic acid, Tween) using a biphase (water and chloroform or hexane) transfer method. It is found that both the structure of the surfactant and the native surface of the ligand govern the coating of the NCs with surfactants. More specifically, the hydrophobicity-hydrophilicity balance of the surfactant regulates the coating efficacy, thereby transferring the NC from the organic to the aqueous phase. The type of ligand on the NCs and the kind of coating surfactant also affect photoluminescence (PL). The ratio of PL and absorbance unit (defined as PL per 0.1 AU) was implemented as a tool to monitor changes in PL intensity and wavelength as a function of size, coatings and surface defects. Finally, the distribution of CdSe nanocrystals between pseudophases in cloud point extraction was discussed based on experimental results. It was concluded that the size of CdSe nanocrystal present in an appropriate pseudophase is correlated with the way in which the non-ionic surfactant coats CdSe nanocrystals.

**Electronic supplementary material:**

The online version of this article (doi:10.1007/s00604-013-1062-z) contains supplementary material, which is available to authorized users.

**Figure:**
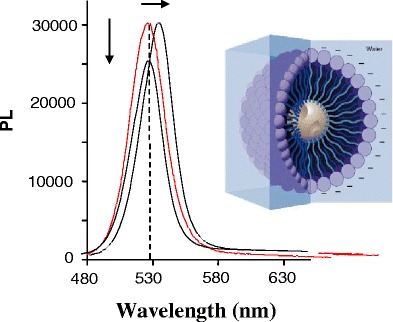
Coating of CdSe semiconductor nanocrystals with surfactants impacts nanocrystals’ spectral features. Absorbance of first exciton absorption band was used to estimate ability of surfactant to disperse CdSe nanocrystals. Photoluminescence (PL) intensity and position of PL band were analysed in terms of nanocrystal’s surface phenomena via surfactants applied for coating.

## Introduction

Colloidal semiconductor nanocrystals (quantum dots) represent one of the most interesting and extensively studied systems due to the quantum confinement effect and size-and surface-dependent electronic and optical properties. Among them, CdSe nanocrystals (CdSe NCs) are one of the most examined due to their simple synthesis and size-controlled bandgap that covers the entire visible spectrum. An additional NC property of interest is related to surface phenomena, between the crystal surface and its attached ligand. The main requirement for this is the stable ligand attachment to crystal surface. In the case of CdX (X = S, Se, Te) NCs, fatty acids, thiolates, and a variety of amine and alkyl derivatives of R_3_P or R_3_P=O have been shown to provide stable surface passivation for dispersion of the NCs into various solvent systems. Those prevent degradation of the NC core, provide surface functionality for subsequent conjugations including bio-conjugation, and provide useful optical signatures specific to the NC-ligand system. These issues and others, including methods for characterizing nanostructures and their applications have recently been extensively reviewed [1–18].

One of the critical requirements in nanomaterial functionalization is precise control of its surface chemistry [2]. However, a large surface area to volume ratio of nanomaterials relative to the bulk increases the overall surface energy of a nanomaterial, thereby increasing its reactivity. Surface chemistry influences the surface energy, functionality, and structural stability of nanomaterials; and as a result, can be used to modulate surface energy that dictates the function of a nanomaterial [2, 3].

Amphiphilic molecules play a useful role in the modification of a surface of a nanomaterial. General aim is the dispersion of nanostructures into aqueous or non-aqueous (e.g. reversed micelles) media and the procedure impacts particle’s hydrodynamic parameters (surface potential, radius [19, 20]), thereby its mobility or diffusivity. Most examples have been shown with carbon nanostructures dispersed in aqueous media [21–24]. Another direction, especially for metallic (Au, Ag) or semiconductor nanomaterials is their characterization and this direction was the center of our research effort [25–28]. As confirmed by capillary electrophoresis amphiphile-coated nanocrystal were shown to transform nanocrystals into an object with a chosen electrostatic character (i.e., anionic, cationic or non-ionic) which also behaved like a micellar entity. It was shown that electrophoretic discrimination of a particle versus a particle-DNA conjugate could be achieved [27], as well as electrophoretic extraction of particles from a matrix [28], or visualization of particles passivated with electrically neutral surface ligands [26].

In the course of many experiments reported thus far [25–28], we have gathered interesting data concerning surface modifications of CdSe nanocrystal and the colloidal behavior of surfactant-modified nanocrystals. These constitute the present discussion framework that addressed four main observed phenomena: (i) dispersion of CdSe NCs using surfactants and impact of this on CdSe nanocrystal photoluminescence (PL), (ii) the shift in position of the photoluminescence (PL) band of CdSe nanocrystals due to surfactant coatings, (iii) features related to coating NCs with oleate surfactant, and (iv) the size-dependent distribution of CdSe nanocrystals between surfactants phases. The latter shows, for the first time, a relationship between NC size and its attachment to a particular phase in cloud-point extraction.

## Experimental

### Reagents

All chemicals and reagents used herein were of analytical grade. For synthesis of the tributylphosphine (TBP) or trioctylphosphine (TOP)-coated CdSe nanocrystals, cadmium oxide (~1 μm, 99.5 %), selenium powder (100 mesh, 99,999 %), tributylphosphine (TBP, 97 %), trioctylphosphine (TOP, 90 %), trioctylphosphine oxide (TOPO, 99 %) from Sigma-Aldrich (St. Louis, USA, http://www.sigmaaldrich. com) or 1-octadecene (ODE) from Across Organics (Morris Plains, USA, http://www.acros.be/) were used. Aqueous solutions of amphiphiles were prepared from ionic or non-ionic surfactants obtained from Sigma-Aldrich, apart from sodium oleate (OA) or sodium laurate (LA) (Spectrum Chemicals, New Brunswick, USA). Structures/names/shortcuts of compounds used for synthesis and modification of CdSe NCs are shown in Scheme S1 (Electronic Supporting Material; ESM).

### Instrumentation

For photoluminescence measurements, a Safire (Tecan Group Inc., www.tecan.com Männedorf, Switzerland) instrument was used. The excitation wavelength was 450 nm and the PL spectra were collected from 480 to 740 nm. UV–vis measurements were conducted with a HP8453 UVVIS spectrophotometer with HP ChemStation software (Hewlett-Packard, http://www8.hp.com, Palo Alto, CA), using a 1 cm cuvette. TEM measurements were conducted on a Hitachi H7000 (Hitachi, http://www.hitachi.com/, Japan) system operating at 90 kV or JEOL JEM-2100 Scanning Transmission Electron Microscope (STEM) (JEOL, http://www.jeol.com, Tokyo, Japan) in particular cases. One drop of a dilute sample of CdSe was placed onto a Formvar coated copper grid, allowed to settle for 20 s, and wicked away using an absorbent tissue. Size analysis was performed on digital images captured with an ImageJ V. 1.34 s (http://rsbweb.nih.gov) program.

### Procedures

#### Modification of nanocrystal surface by surfactants

Procedures for synthesis of CdSe nanocrystal were posted in ESM. Samples of surfactant coated CdSe NCs were prepared by mixing 50 μL of CdSe NCs dispersed in organic solvent (chloroform, hexane; c_NC_ ~ 10^−5^ M) with 300 μL of surfactant solution. The mixture was left to stand overnight at room temperature, in the dark area, stirring with a magnetic stir bar to evaporate the solvent. Such prepared samples, before particular experiments, were centrifuged (15,000 rpm/10 min) to remove aggregates. Details of the composition of a surfactant solution appear in the figure captions.

## Results and discussion

### Dispersion of CdSe NCs in surfactants

Absorption (UV–vis) and photoluminescence (PL) are basic optical techniques for characterizing semiconductor nanocrystals based on their unique spectral characteristics. For CdX (X = S, Se and Te), the position and intensity of the first exciton band can be used to determine the size of the NC core as well NC solution concentration. The original method using UV–vis technique in this manner was recently reiterated [29, 30]. In the present work the transfer of CdSe NCs from organic to aqueous phase, as aided by coating NCs with selected surfactants using biphase transfer method (graphical interpretation see Fig. S1, ESM), was applied to determine the effects of the surfactant molecular structure. For this, the position and intensity of the first exciton band was used to monitor the transfer in terms of the size and concentration of nanocrystals. This approach was expanded to include the ratio of PL per normalized NCs concentration (i.e., PL/0.1 AU), to observe changes in the electronic interaction between CdSe nanocrystal and a surfactant. Further details can be found in the ESM (Figs. S2-S3).

### Transfer of CdSe NCs from organic to aqueous phase mediated by surfactants

Fig. S4 (ESM) shows an example of the transfer of CdSe NCs using sodium oleate surfactant (OA). Based on the figure, it can be stated that there is a minimum threshold of surfactant concentration to provide dispersion of the CdSe NCs into aqueous solution, below which aggregation and precipitation occur. It was concluded that ability of surfactants to transfer CdSe NCs from organic to aqueous phase follows the order: cationic > anionic > non-ionic, in agreement with the previous work [28].

**Fig. 1:**
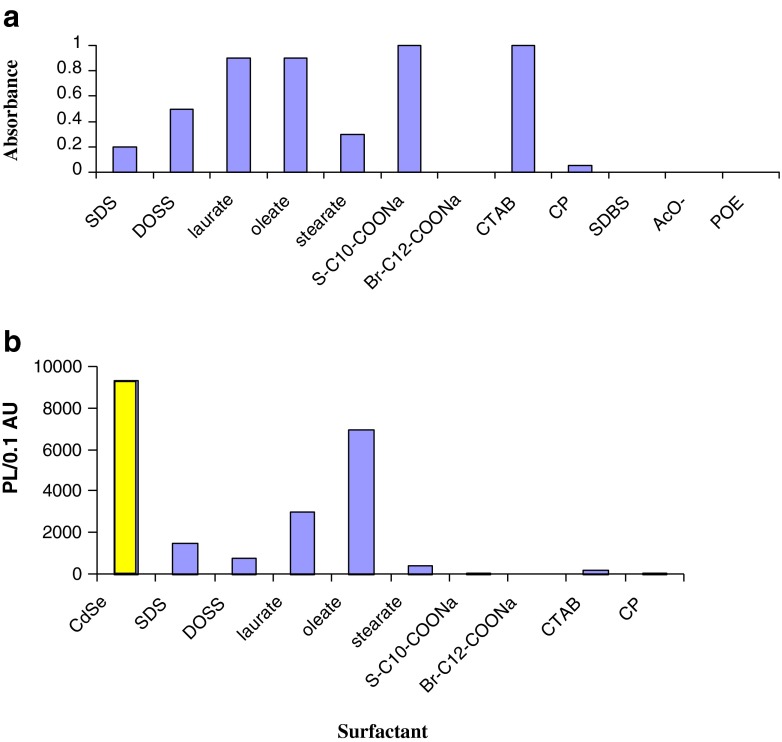
Absorbance (*first exciton band*) of aqueous solution of CdSe NCs due to NCs transfer from organic to aqueous phase by means of appropriate surfactant (frame **a**). Data on average for CdSe NCs size interval 2.9–3.3 nm. Sample preparation: 50 μl chloroform solution of CdSe NCs 2–5 × 10^−6^ M was dispersed in 300 μl of surfactant solution. After overnight coating samples were centrifuged before optical measurement. Concentration of surfactants 100 mM apart from stearate and HS-C_10_-COOH (both 50 mM). Note, that coating with non-ionic surfactants affords for limited transfer (sample absorbance < 0.3; see Fig. S11, ESM). Frame **b**, PL efficiency per normalized CdSe NCs concentration (PL/0.1 AU) for ionic surfactants and starting CdSe NCs dispersed chloroform (yellow bar). Samples are prepared according to frame **a**

The type of surfactant capable of facilitating stable NCs solutions in water was further explored to elucidate contributions from hydrophobic tail groups and hydrophilic head groups (Fig. 1). The first issue is a role of the hydrophobic part of the surfactant. By comparing CH_3_COO^-^ (AcO^-^) vs. R-COO^-^ surfactants (OA, LA) or a non-ionic surfactant vs. poly(ethylene)oxide (PEO) chain, it can be stated that dispersion of NCs was only observed in surfactants solutions. This means that the presence of a hydrophobic hydrocarbon chain on the surfactant is necessary for effective coating of CdSe NCs. This is in agreement with ref. [31], where authors claimed that PEG (HO(CH_2_CH_2_O)_n_OH) did not allow dispersion of CdSe NCs. It is important to note that CdSe NCs were initially capped with hydrophobic surface ligands (i.e., TOP or TOPO) in the course of NCs synthesis. Thus, as-prepared CdSe NCs should only be water soluble when coated with an amphiphilic surfactant with its hydrophobic portion bonding through van der Waals interaction with the NC surface ligand, allowing solvation by the hydrophilic headgroup of the surfactant molecule. However, dispersion of NCs into aqueous solvents requires consideration of both the head and tail group of a surfactant, where it was found that some headgroups are less effective in providing water dispersion (e.g., Igepal CO-201). As well, the tail group of some surfactants was less effective in van der Waals associations with the parent NC ligands. These results indicate a mixed mechanism for the efficacy of coating CdSe NCs with surfactants.

**Fig. 2:**
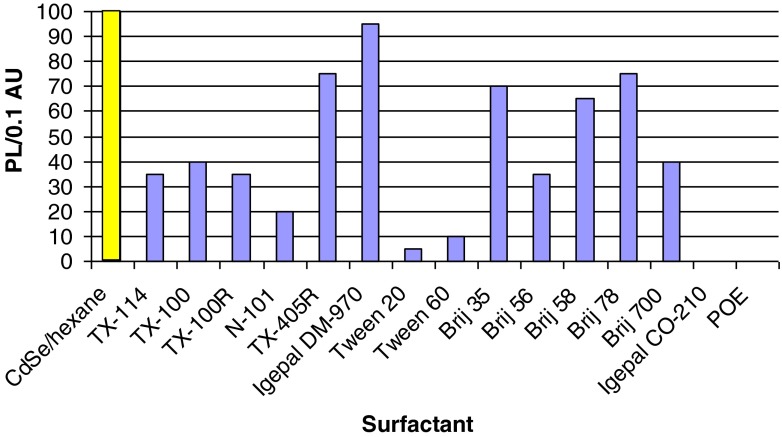
PL efficiency per normalized CdSe NCs concentration (PL/0.1 AU) using non-ionic surfactants for coating. NCs with size 3.5–3.7 nm were used. Concentration of non-ionic surfactants were 5 % (w:w), apart from TX-100 and TX-114, where the highest PL/0.1 AU ratio was obtained using 2.5 % w:w surfactant solution. NCs dispersed in hexane were used. In the case of Tween 20 and 60 surfactants, NCs aggregation was seen using hexane. Slight NCs dispersion in Tween surfactants was obtained using solution of NCs dissolved chloroform

Therefore, by comparing cationic surfactants (CTAB vs. CP) or anionic (SDS, DOSS vs. SDBS), it can be stated that ionic surfactants containing ring structures (CP, SDBS) were unable disperse and transfer CdSe NCs to an aqueous phase due to negligible coating, which led to NC aggregation and precipitation. A similar behavior was previously reported for CHAPS {3-[(3-cholamidopropyl)-dimethylammonio]-1-propanesulfonate} or sodium chelate [28]. Interestingly, such behavior is in contradiction to carbon nanotube/surfactant systems, where the SDBS surfactant was found to be the most efficient coating agent. Inspection of Fig. 2 posted in next section allows to state that coating of CdSe NCs with branched surfactants is inactive (see Tween surfactants). It is worthy to note, that Tween 60 was found to be the best dispersing agent for carbon nanotube among tested non-ionic surfactants [32]. Also, by comparing R-COO^-^ surfactant (LA, OA) vs. Br-C_12_-COO^-^ it can be stated that the presence of non-binding atom (e.g., Br) at the end of tail group of the surfactant hydrocarbon chain negates van der Waals interactions of the surfactant with the hydrophobic NC surface ligand and dispersion in water is not observed. Both examples (Tween, Br-C_12_-COO^-^) lead to the conclusion that second-order phenomena (e.g. lying down of surfactant chain on a nanocrystal) can affect interactions between CdSe NCs and surfactants. Also, the Tween example shows a possible effect related to solvents used for dispersing CdSe NCs (Fig. 2). The solvent effect for CdSe was already discussed in literature [33], specifically for NC optical spectra [30].

To support the discussion upper, molecular modeling at the DFT level was applied to analyze situations observed experimentally (Table S1, ESM). Taking into account the most important faces of CdSe nanocrystal ([110], [001] and [00–1]), the calculated binding energies between a particular facet and a ligand support the observed binding order for surfactants: cationic (−NR_4_^+^) > anionic (−COO^−^) > non-ionic. A more complete modeling analysis should allow for distinguishing between binding and non-binding surfactants (see graphical interpretation Fig. S5, ESM). Such sub-classification of surfactants was observed experimentally, i.e., surfactant able to coat a nanocrystal via its head group vs. surfactant unable to do this (e.g. R-COO^-^ vs. R-(O)SO_3_^-^) [28]. Also, ref. [34] distinguishes reversibly and irreversibly bound ligands. In such a situation, pyridine (py) ligand can serve as the threshold for these both groups of surfactants, due to remarks posted in the previous paper [28]. Taking into account the aforementioned threshold it can be stated that anionic surfactants can be divided into binding (OA, LA) or non-binding (SDS, DOSS). Such sub-classification of surfactants was observed experimentally. An interesting example can be a relative coating of CdSe NCs with CTAB surfactant (Fig. S6, ESM). Based on this it can be stated that for CdSe NCs passivated by TOPO/TBP, CTAB behaves as a binding, whereas for NCs passivated by TOPO/TOP ligands as non-binding ligand, respectively.

Examples of optical characterization of the TOPO/TBP and TOPO/TOP passivated CdSe NCs is presented in the ESM. Figure S7 shows spectra (UV–vis and PL) for two types of CdSe NCs coated with surfactants. It was confirmed that position of PL wavelength does not depend on NC concentration. Figures S8 and S9 address the issue of NC etching (reduction in the CdSe core diameter) from surfactant coatings. It was observed that, among the tested surfactants, non-ionic Triton N-101 surfactant does in fact etch CdSe nanocrystal. This can be of practical significance as a tool for estimating the quality of CdSe NCs and its degree of passivation.

### Electronic coupling between CdSe NCs and surfactants

According to ref. [1], surface ligands for CdSe semiconductor nanocrystals able to withdraw electrons from a nanocrystal decreases photoluminescence, whereas ligands that can donate electrons to CdSe NCs can stabilize the high photoluminescence. In other words, if a ligand is able to separate the (hole–electron) pair a decrease in PL of nanocrystal is observed. With this in mind, various ligands conditions were tested [1, 2, 33, 34]. In the present work, PL per normalized NC concentration (PL/0.1 AU) was used to discuss PL changes in CdSe NCs due to coating with surfactants. The tool was discussed in details in ESM, Figs. S2-S3 and is based on the defects-free crystal and PL constancy over similar size nanocrystals (e.g. 2.4–3.7 nm). Based on the tool and taking Fig. 1b and 2 into account, it can be concluded that the order in PL intensity is opposite to efficacy in NCs transfer and follows the order of decreasing PL/0.1 AU, i.e.,: non-ionic > anionic >> cationic surfactants.

The electronic interaction was observed to be the most significant for non-ionic coating surfactants and this group of surfactants was analyzed more deeply. Figure 2 shows results gathered due to coating with non-ionic surfactants. Taking into account surfactant molecular structures (Scheme 1, ESM) and Fig. 2, the contribution of a particular portion of a surfactant to a change in PL can be estimated. The following features were compared: (i) the same hydrocarbon skeleton vs. different length of poly(ethylene) oxide chain (Igepal CO-210 vs. TX-100 or TX-100R vs. TX-405R or Brij 56 vs. 58 or Brij 78 vs. 700); (ii) the same PEO chain vs. different hydrocarbon skeleton (TX-100 vs. TX-100R or Brij 58 vs. 78), and (iii) comparison of non-ionic surfactants due to small structural changes (TX-100 vs. N-101). At least three general conclusions are available from these examples. First, the presence of a short ethylene oxide chain (Igepal CO-210) did not result in NCs dispersion. Second, longer poly(ethylene oxide) chains of a surfactant generates higher PL intensity (TX-405R, Brij 58, 78 and DM-970) and third, comparing surfactants with the same poly(ethylene) oxide chain (TX-100 vs. TX-100R (*n* = 10) or Brij 35, 58 or 78 (*n* = 23 or 20)) vs. different hydrocarbon skeleton, a similar PL efficiency was seen for each group of surfactants. From these deductions, it can be stated that a balance between hydrocarbon and poly(ethylene) oxide moiety of a surfactant defines the role a surfactant, in altering the NC photoluminescence, during the coating process. For example, the PL/0.1 AU increases in the following order for PL intensity: Igepal CO-210 < < Brij 700 < Brij 78 < DM-970, where the latter affords the highest electronic interaction with the highest PL efficiency, due to appropriate balance between hydrocarbon and ethylene oxide moieties of a surfactant. It should be noted, that Tween surfactants show that the system can be more complex.

The dispersion of CdSe NCs using non-ionic surfactants allows one to examine the usefulness of the PL/0.1 AU tool over range of CdSe nanocrystals sizes (Fig. S10, ESM). In the experiment, it was found that for the sample of CdSe NCs with lower PL efficiency, coating with non-ionic surfactants restores nanocrystal PL yield to expected values. This indicates that surface defects, which cause non-radiative carrier recombinations, were eliminated by coating with these surfactants, despite the change in NCs environment from organic to aqueous.

In order to highlights issues discussed above, features (spectra UV–vis, PL and PL/0.1 AU ratios) are presented in the ESM (Fig. S11, ESM) for selected non-ionic surfactants.

### Bathochromic shift in λ_max_ of photoluminescence (PL) for semiconductor CdSe nanocrystals with the same energy gap (E_g_)

**Fig. 3:**
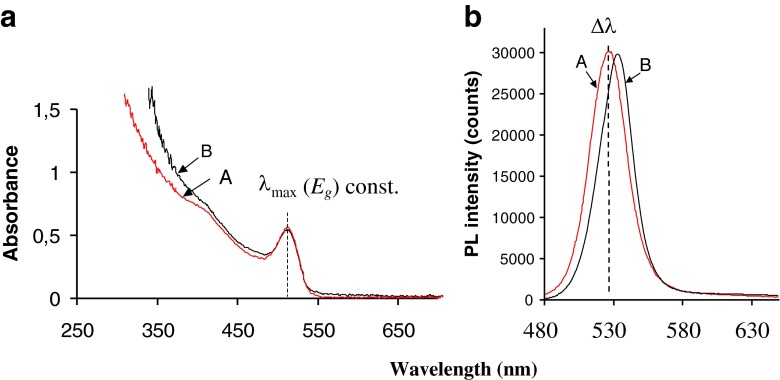
Bathochromic shift in λ_max_ of photoluminescence (PL) for semiconductor CdSe nanocrystals with the same energy gap (E_g_). Semiconductor nanocrystal CdSe and its (**a**) absorption UV–vis and (**b**) photoluminescence (PL) spectra. Notation: **A**, nanocrystal CdSe, core size 2.6 nm, obtained from tri-n-octylphosphine oxide synthesis and dispersed in hexane; **B**, the same NC after ligand exchange with carboxylic acid ligand/dispersion in water

Figure 3 shows an example of the red-shift observed in the PL spectra for CdSe nanocrystals with the same position of first exciton band as measured by UV–vis absorbance.

Characteristic band in absorption spectrum (λ_max_; *first exciton band*) reflects quantum confinement effect [refs. 29, 30] and energy gap (*E*_*g*_), as well. Typical effect due to a ligand exchange is constant Stokes shift (λ_em_ – λ_Abs_) for nanocrystals with the same core dimension. In particular situations an exchange of a surface ligand or coating (**A** vs. **B,** Fig. 3) lead to bathochromic shift in the position of λ_em_(PL), despite the same λ_max_(Abs). The term Δλ denotes the difference between Stokes shift **A** vs. **B**. The shift in the PL λ_em_ maxima was attributed to delocalization of the exciton to the surfactant coating. A similar effect was recently reported for thiol capped CdSe NCs [35].

A further illustration of this phenomenon is shown in Figure S12 (ESM) for 2.5 nm CdSe NCs synthesized with either TOPO/TBP or TOPO/TOP surface ligands. As shown, the emission maximum shifts by 11 nm between TBP vs. TOP coating. The Δλ_em_ diminishes as NC core diameter increases and above a core size of 3 nm, no shift was observed. Concentration effects (aggregation or photoluminescence self-quenching) were ruled out (spectrum A vs. A’; frame **b** Fig. S12, ESM) as the cause for the shift in λ_em_.

**Fig. 4:**
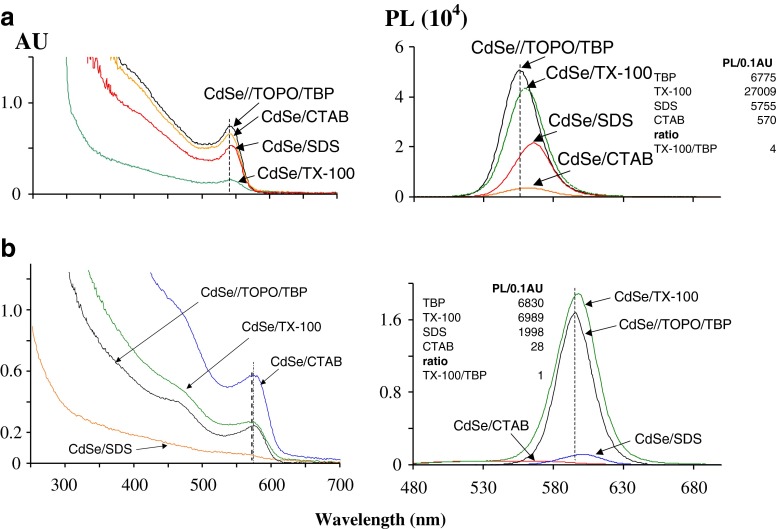
Coating of CdSe nanocrystals with surfactants. UV–vis spectra (left column) and photoluminescence spectra (right column). CdSe size: frame **a** 2.9 nm; frame **b** 3.7 nm. Samples: CdSe//TOPO/TBP NCs dispersed in chloroform and these NCs dispersed in aqueous solution of: CTAB; SDS (both 100 mM) and TX-100 (5 % w:w). Inserts frames **a** and **b** contain PL/0.1 AU for each sample, as well as ratios in PL/0.1 AU for TOPO/TBP vs. TX-100 coating, which defines an extend of the reconstruction of the surface of CdSe//TOPO/TBP nanocrystals due to coating with TX-100 surfactant

A similar spectral shift in λ_em_ (PL) vs. fixed position of λ_max_ (Abs) was observed for CdSe NCs coated with either ionic or non-ionic surfactants. In these cases, PL intensity for NCs follows the order: non-ionic > anionic >> cationic surfactant used for coating (Fig. 4). This is in agreement with Figs. 1b and 2. Although, a red shift in λ_em_ (PL) for nanocrystals with the same λ_max_ (UV–vis) was observed for all types of surfactants examined here (Fig. 4, frames **a**, **b**), non-ionic surfactant was further examined due to observed the highest PL/0.1 AU ratio according to Fig. 2. As an example, the non-ionic surfactant Triton X-100 (TX-100) was used. Frames **a**, **b** of Fig. 4 enable to establish the relationship between: NC size, degree of PL spectral shift and PL intensity. Note, that the accompanying action is NC transfer from organic to aqueous phase. By comparing NC size (Fig. 4, frame **a**, **b**), it can be concluded that for smaller NCs, the PL efficiency is related to the extent of the PL red shift vs. fixed λ_max_ (Abs). In this situation, the ratio in PL/0.1 AU for smaller NCs coated with TX-100 is approximately four times greater than that for larger NCs coated with TX-100 (see insets of frames **a** and **b** of Fig. 4). However, according to Fig. S10 (ESM) and the discussion of Fig. S2 and S3 such a ratio, should be closer to 1.5 based solely on NC size. A similar conclusion can be drawn by comparing Δλ (λ_em_-λ_Abs_) vs. PL efficiency for CdSe//TOPO/TBP vs. its TX-100 derivative, considering smaller vs. bigger CdSe NCs (Fig. 4). In this situation data for smaller NCs (Δλ = 10 nm, ratio in PL efficiency (TX-100 vs. TOPO/TBP) = 4) vs. data for bigger NCs (Δλ = 0 nm, ratio in PL efficiency (TX-100 vs. TOPO/TBP) = 1) allows one to conclude that the crystal surfaces of a smaller NCs are more affected by choice of surface coating with surfactants than bigger NCs. This phenomenon and its consequence are discussed in following two Sections.

It is worth nothing that this observed shift in λ_em_ (PL) vs. fixed λ_max_ (Abs) may be associated with other phenomena as yet determined. An analysis of available literature references on this issue is posted in ESM (page 18). From this, it can be concluded that a relationship between Δλ and PL efficiency, as presented here, have not been previously reported.

### Coating with oleate surfactant (OA)

CdSe NCs passivation with oleate surfactant was recently analyzed in details [36] and the interaction between a crystal surface and oleate surfactant was discussed in terms of the coating mechanism. Our preliminary results (Fig. S13, ESM) show bright photoluminescence under UV light after coating NCs with OA. As with most cases examined in the present work, OA was used to facilitate the transfer of CdSe NCs from organic to aqueous solution phases. The PL/0.1 AU tool was used to analyze three different NC sizes (Fig. S14, frame **a**, 2.6 nm, **b**, 3.1 nm and **c**, 5.1 nm) coated with OA. As above it was observed that only smaller NCs coated with OA produce a high PL efficiency when a shift in λ_em_ (PL) occurs. For bigger NCs, despite the occurrence of a PL spectral shift, the PL efficiency was much lower than the uncoated NC starting material of the same size.

**Fig. 5:**
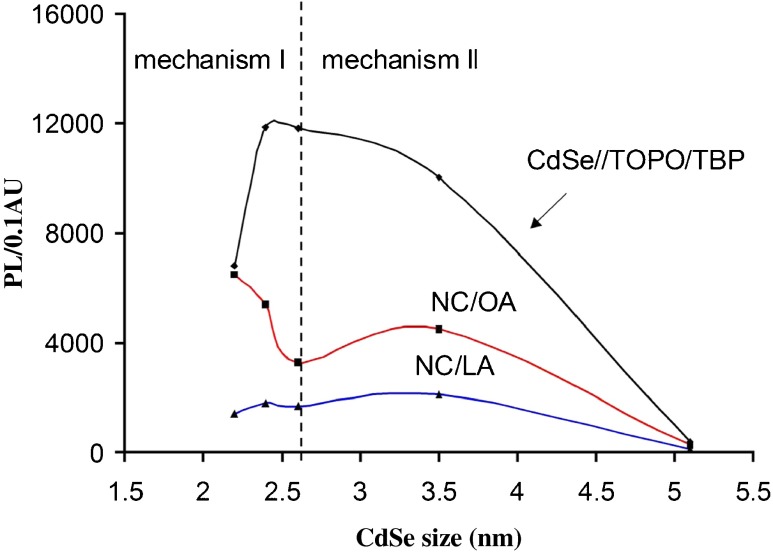
Coating of CdSe//TOPO/TBP NCs with (LA, OA) surfactants in terms of PL/0.1 AU for CdSe nanocrystals about different core sizes. Black line represents PL/0.1 AU factor for CdSe//TOPO/TBP NCs dispersed in organic solvent. Respectively, red and blue lines represent these CdSe nanocrystals dispersed in aqueous solution of OA or LA surfactants, respectively. Note that OA (pure) and LA are non-fluorescent molecules

This aspect was further analyzed in terms of PL/0.1 AU factor over various NCs sizes as shown in Figure 5. As can be seen, the starting material CdSe//TOPO/TBP has two regions that illustrate the PL response. Region I defines NCs of this type that are < 3 nm, where a decrease in PL/0.1 AU can be explained by the presence of surface defects. In this region the PL/0.1 AU response for OA vs. LA coated NCs is not the same and only in the case of OA-coated NCs did the PL/0.1 AU factor increases with a decrease in NC size. Therefore, oleate surfactant can serve as a tool for eliminating surface defects related to small CdSe NCs. When considering the structural differences in LA vs. OA (structures posted in Scheme 1, ESM) the presence of –C=C– group of OA surfactant is the most important difference. It should be noted that, in comparison, the stearate surfactant did not provide high PL efficiency (Fig. 1b or S 13) which shows that the saturated hydrocarbon chain is not coupled with the NC surface. Therefore, the presence of the –C=C– group in OA structure is the source for the effect. Such an effect was previously reported for olefins and an increase in PL of CdSe crystal was reported [37]. Also, the theoretical model (Table S1, ESM) confirms an increase in binding energy of a molecule, used for CdSe crystal coating, due to the presence of –C=C– groups in a capping molecule. Therefore, it can be stated that the PL/0.1 AU increase is only observable with surfactant containing –C=C– groups and for CdSe NCs ≤ 2.5 nm.

### Cloud-point extraction for CdSe nanocrystals dispersed in TX-114 surfactant

**Fig. 6:**
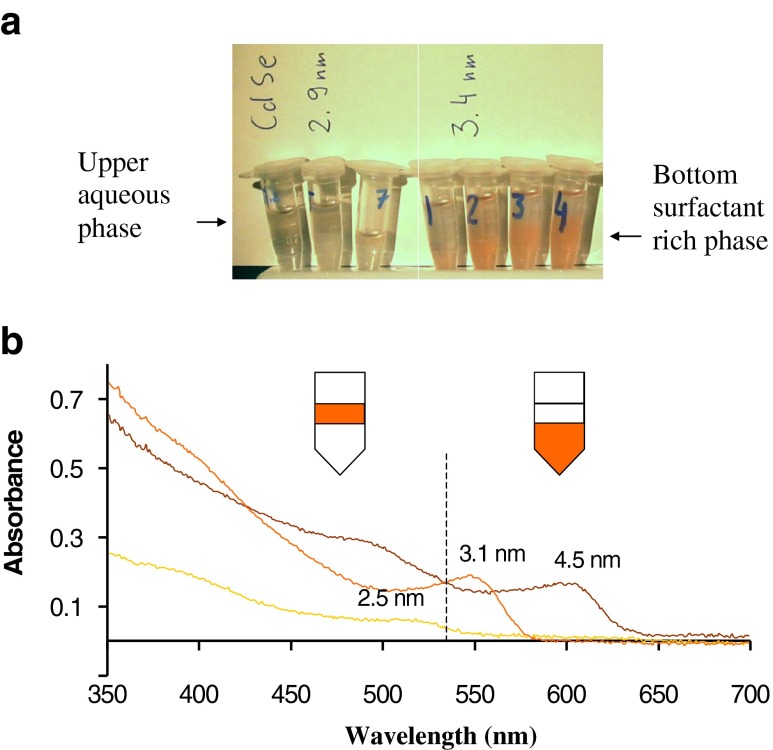
(frame **a**) Distribution of CdSe//TOPO/TOP nanocrystals, according the nanocrystal core size, between upper and bottom phases using cloud-point separation. In the experiment, the presence of bigger CdSe NCs (3.4 nm) in bottom, as well as the presence of smaller NCs (2.9 nm) in upper phase were seen, irrespective on the concentration of TX-114 used for extraction procedure. Frame **a**, vials labeled 1–4 right-hand side, contain 2.5, 5, 7 or 10 % (w:w) TX-114, respectively. Respectively, vials left-hand side contain 2.5, 5 and 7 % (w:w) TX-114. Frame **b:** UV–vis spectra for upper and bottom phases due to cloud-point separation of CdSe//TOPO/TOP NCs with the use of TX-114 surfactant. Either 4.5 or 3.1 nm CdSe NCs was present in the bottom surfactant rich layer, whereas smaller 2.5 nm CdSe NC was found in the upper layer, as confirmed by UV–vis spectra. Samples preparation (frame **b**): NCs in hexane were stirred overnight with 5 % w:w TX-114 solution, followed by centrifugation (8,000 rpm/8 min) to remove aggregates. Such obtained samples were slightly heated until turbidity, followed by sample centrifugation

Cloud-point extraction, using non-ionic surfactants, is an important analytical technique for enrichment of variety of substances [38]. In this technique a micellar solution of a non-ionic surfactant above the cloud-point temperature separates into two isotropic phases: (i) a surfactant rich phase and (ii) an aqueous phase containing surfactants at the critical micelle concentration. For the TX-114 surfactant, the cloud point is at 22^°^C [38], which is suitable to study nanocrystals passivated by ligands. In the present experiment, CdSe nanocrystals obtained according to TOPO/TBP or TOPO/TOP synthesis were dispersed in aqueous solution of TX-114 and the obtained solution was heated slightly above 22^°^C until turbidity was observed. After centrifugation, two phases (upper aqueous phase and lower surfactant rich phase) were obtained (Fig. 6 frame **a**). UV–vis spectrophotometric measurement of both phases shows that CdSe nanocrystals dispersed in TX-114 with core size < 3 nm were present in upper phase, whereas these with > 3 nm were present in bottom phase (Fig. 6, frame **b**). It should be noted that CdSe NCs obtained according to the ODE/OA synthesis are unsuitable for the cloud-point extraction due to the negligible dispersion in aqueous TX-114 (Fig. S7, ESM).

The concentration of TX-114 used to perform cloud-point extraction was in the range of 2.5–10 % (w:w). The distribution of NCs between phases was found to be independent of the TX-114 concentration (Fig. 6, frame **a**).

**Fig. 7:**
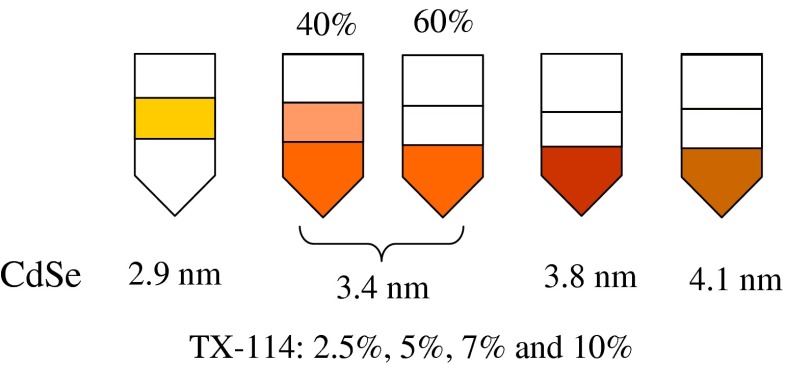
Distribution of CdSe nanocrystals, according to their core sizes, using cloud-point extraction. Samples of CdSe//(TOPO/TBP) in hexane were dispersed in aqueous solutions of TX-114 (range: 2.5–10 % w:w for each NC) during ca. 12 h (overnight). Next, samples were centrifuged (8,000 rpm/8 min) to remove aggregates. Such obtained samples were used to cloud-point extraction procedure. In the case of CdSe  3.4 nm, regarded as threshold, two possibilities were observed, namely CdSe NCs present in both layers simultaneously (ca. 40 % of instances) or CdSe NCs present only in bottom layer (ca. 60 % of instances)

A slightly different threshold of crystals size of 3.0 nm (TOPO/TOP) vs. 3.4 nm (TOPO/TBP) for NC present in upper vs. bottom phase was observed using CdSe nanocrystals obtained from (TOPO/TBP) synthesis (Fig. 7). As above, under such conditions the distribution of CdSe nanocrystals, according to their sizes, was independent of TX-114 concentration over the range of 2.5–10 % (w:w).

For the CdSe/TX-114 system, UV–vis spectrophotometric measurements revealed that small CdSe NCs (< 3 nm core size) are exclusively present in upper phase with no trace of NCs in the bottom phase, irrespective of TX-114 concentration (2.5–10 % w:w). This situation was analyzed in terms of PL/0.1 AU (Fig. S15, ESM). There are two conclusions from the experiment (Fig. S15, ESM). First, PL efficiency decreases with increase in TX-114 concentration and secondly, PL efficiency is directly related with red shift in λ_max_ (PL), in agreement with discussions above.

**Fig. 8:**
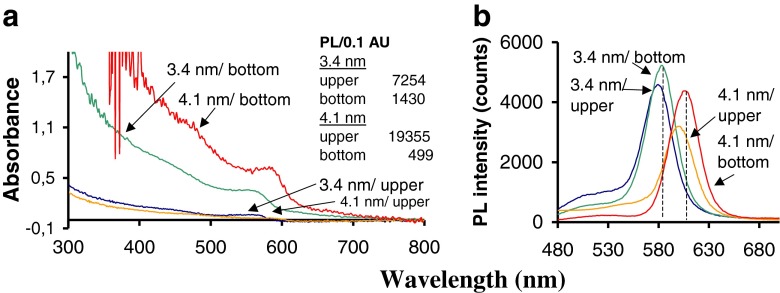
Spectral features for CdSe NCs size > 3 nm present in upper and bottom layer due to cloud – point extraction for CdSe NCs dispersed in TX-114 solution. The first example (3.4 nm NC) reflects the presence of CdSe NC in both layers simultaneously and the second case is the example of CdSe NC (4.1 nm size) present mainly in bottom layer (see Fig. 7). Insert shows the ratio PL/0.1 AU for each CdSe nanocrystal in each layer. The spectral features and PL/0.1 AU ratio for the staring materials (CdSe//TOPO/TBP in hexane): 559 nm/584 nm (λ_max_, UV–vis/λ_em_, PL) and PL/0.1 AU 3,900 for 3.4 nm CdSe nanocrystal, and 588 nm/604 nm and 13,000 for 4.1 nm NC, respectively

A similar PL/0.1 AU analysis was done for bigger CdSe nanocrystals (> 3 nm core size) present in bottom layer of two phases system. Nanocrystals (3.4, 3.8, or 4.1 nm core size), examined above (Fig. 7) were applied. The first conclusion is that CdSe NCs with size > 3 nm are present in both phases, irrespective of the NC examined. In the case of 3.4 nm (Fig. 7), the ratio in NC concentration (bottom vs. upper) reaches 6 in 40 % of the cases, whereas for bigger NCs (3.8 or 4.1 nm), the ratio is greater than 20. Spectra, for upper and bottom phases containing 3.4 or 4.1 nm CdSe NCs respectively, in terms of PL/0.1 AU factor were examined (Fig. 8). It was observed that PL/0.1 AU factor is much smaller for these both NCs present in bottom phase compared to the factor for NCs present in upper phase (see Figure 8 inset).

The first conclusion from the experiment is the observed shift in λ_max_ (PL) for each CdSe NC present in a particular phase (upper vs. bottom), which denotes that NC in each phase are coated differently. Second, PL/0.1 AU factor for CdSe NCs in upper phase, irrespective of the CdSe size, is much higher than this measured for the starting CdSe NC (CdSe dispersed in hexane), which means the deep reorganization of the crystal’s surface in this case.

The main conclusion from experiments discussed above is related to the NC size, its presence in the particular phase, and the PL/0.1 AU factor. These relationships suggest that a distribution of CdSe NCs is due to CdSe NCs different coating of crystal’s surface with the non-ionic surfactant. The picture for CdSe NCs present in upper phase, regardless of the NC size, is effective surface passivation by the non-ionic surfactant and absence or masking of original surface ligands (TOPO, TBP). In other words, the dominant behavior of modified NC is its high polarity that allows NCs to reside in upper aqueous phase. Also, the higher PL/0.1 AU factor than this measured for original CdSe//TOPO/TBP NCs (in hexane) denotes that surface states responsible for non-radiative carrier recombination were removed in this situation. A similar effect was discussed above for results presented in Figure S10, ESM. On the other hand, for bigger CdSe NCs (size > 3 nm) their presence in the surfactant-rich, bottom phase, is because of the NC’s higher hydrophobicity. In this case, the crystal’s surface architecture after coating exposes original surface ligands (TOPO, TBP). This retains the NC hydrophobicity, thereby forces NCs to be present in bottom surfactant phase. In this case, PL/0.1 AU factor is low, which means lack of a surface reconstruction or even increase in number of the surface defects. Both situations are graphically interpreted as seen in Fig. S16 of the ESM.

## Electronic supplementary material

Below is the link to the electronic supplementary material.

ESM 1 (DOC 19473 kb)
