# Supplementary material for: Comparative study on coating CdSe nanocrystals with surfactants
Source: Mikrochim Acta. 2013 Aug 8;180(13):1341–50. doi: 10.1007/s00604-013-1062-z (PMC3779019; doi:10.1007/s00604-013-1062-z)
Supplement: Supplementary file 1 — (DOC 19473 kb) [file 604_2013_1062_MOESM1_ESM.doc]

## Electronic Supplementary Material

# **Comparative study on coating CdSe nanocrystals with surfactants**

Sławomir Oszwałdowski^a^*, Kenneth P. Roberts^b^

*^a^Warsaw University of Technology, Faculty of Chemistry, Department of Analytical Chemistry, ul. Noakowskiego 3, 00-664 Warsaw, Poland*

*^b^Department of Chemistry and Biochemistry, The University of Tulsa, Tulsa, OK 74104, USA*

*Corresponding author: slaosw@ch.pw.edu.pl

## Scheme 1

**Structures of surface ligands and surfactants discussed in the present work**

# Surface ligands

Tributylphosphine Trioctylphosphine


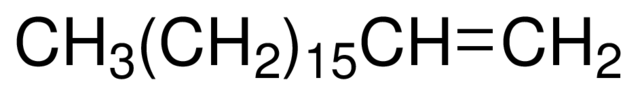
 **TBP TOP**

Pyridine 1-octadecene Trioctylphosphine oxide

**py ODE TOPO**

# Ionic surfactants

**DOSS SDS**

dioctyl sulfosuccinate sodium salt sodium dodecylsulfate

Lauric acid sodium salt **(LA)**

Oleic acid sodium salt **(OA)**


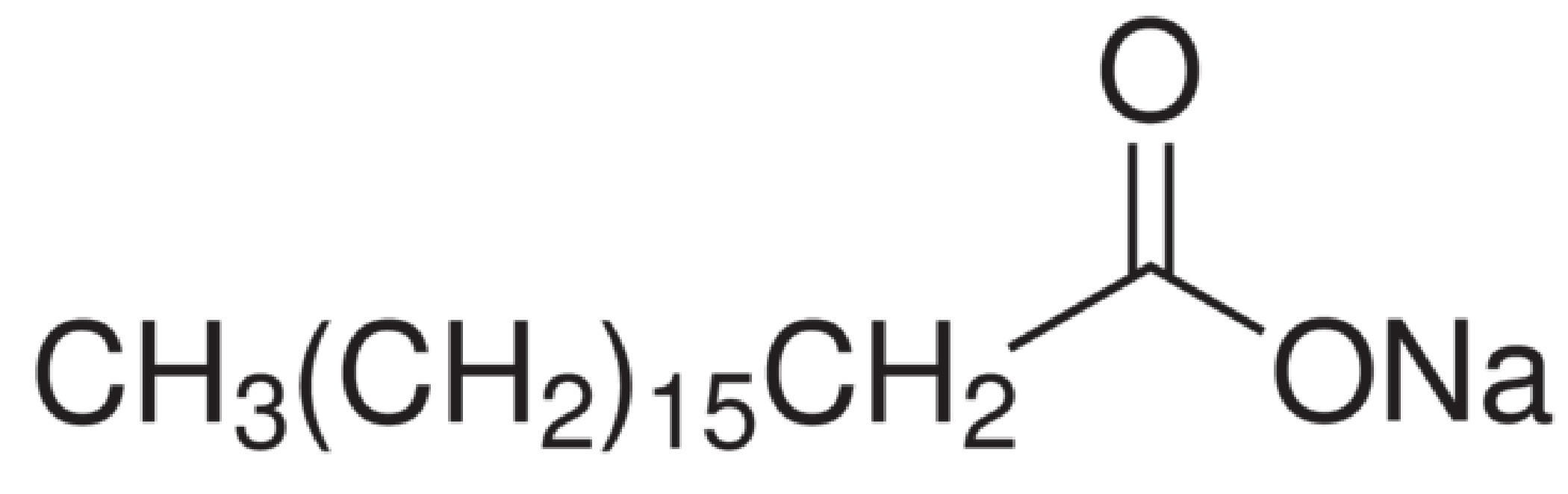
sodium stearate **(SS)** Sodium dodecylbenzenesulfonate (**SDBS**)

##

**Hexadecyltrimethylammonium bromide Hexadecylpyridinium chloride monohydrate** **CTAB CP**

#
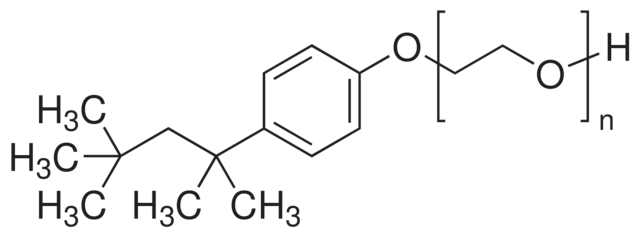
Non-ionic surfactants

n = 9-10 n = 9-10

**Polyethylene glycol *tert*-octylphenyl ether Polyoxyethylene branched nonylphenyl ether**

**TX-100** **N-101**

n = 10

**Polyoxyethylene (10) isooctylcyclohexyl ether Polyoxyethylene branched nonylcyclohexyl ether**

**TX-100R** **N = 101R**

n= 7-8 n = 40

**Polyethylene glycol *tert*-octylphenyl ether Polyoxyethylene (40) isooctylcyclohexyl ether**

**TX-114 TX-405R**

**
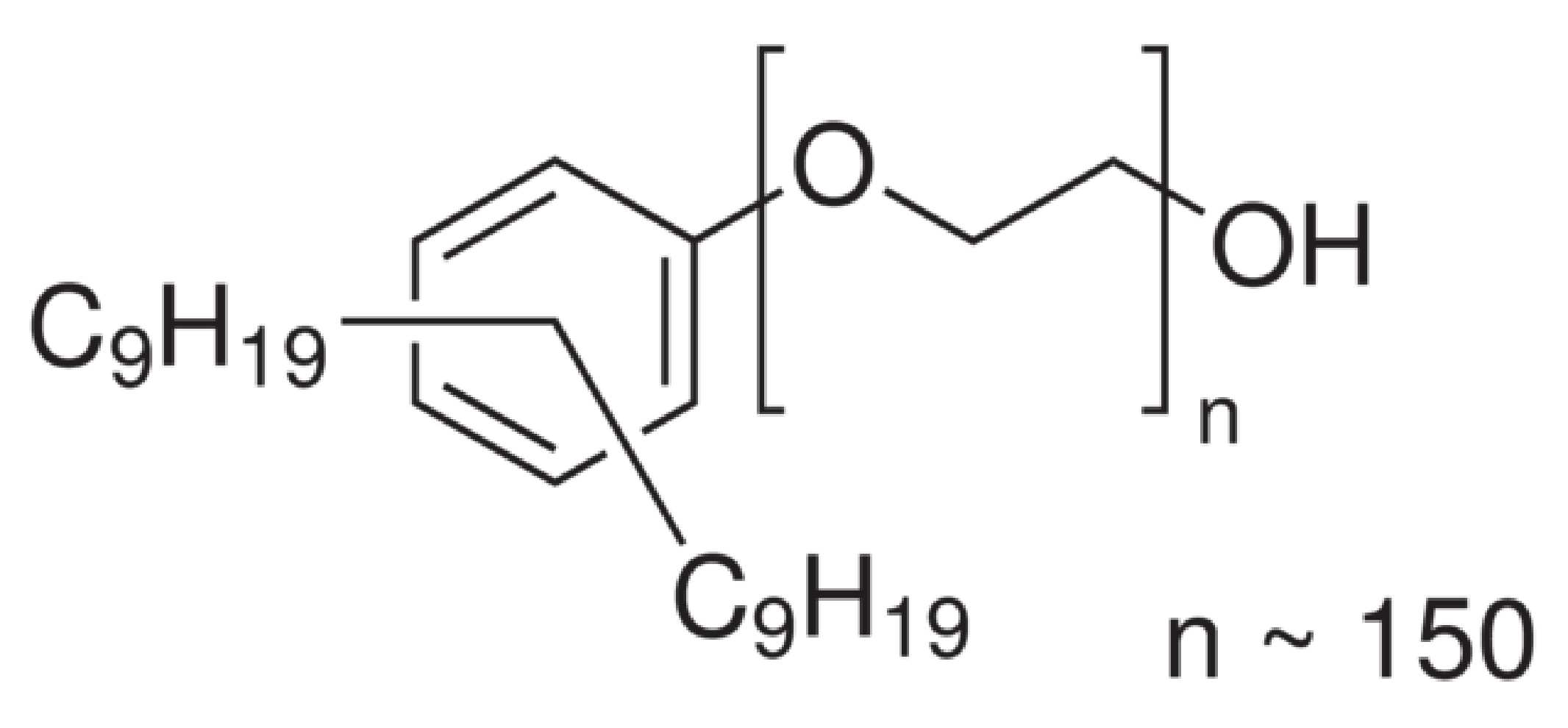
**

n~1.5

polyoxyethylene (2) nonylphenyl ether **Polyoxyethylene (150) dinonylphenyl ether**

**Igepal CO-210 IGEPAL DM-970**

Brij surfactants:

**C_12_E_23_, Polyoxyethylene (23) lauryl ether**

**Brij 35**

C_16_H_33_(OCH_2_CH_2_)_n_OH

**Brij 56** n = 10 **Polyoxyethylene (10) cetyl ether**

**Brij 58** n = 20 **Polyoxyethylene (20) cetyl ether**

**Polyethylene glycol octadecyl ether (n = 20)**

**Brij 78**

**Polyethylene glycol octadecyl ether (n = 100)**

**Brij 700**

###### Tween surfactants

Concerns structures below: **w+x+y+z =20**

**Polyethylene glycol sorbitan monostearate**

**Tween 60**

**Polyethylene glycol sorbitan monolaurate**

**Tween 20**

Reference molecules:

Poly(ethylene) oxide (PEO examined, average M_v_ 600,000 and average M_v_~8,000,000**)**

## PEO

CH_3_COO^-^

Acetic acid anion **(AcO^-^)**

**Procedures**

*Preparation of TOPO coated CdSe nanoparticles*

Hydrophobic TOPO-coated CdSe nanoparticles were prepared from cadmium oxide and elemental selenium [1]. Briefly, the selenium precursor was prepared by combining elementary selenium and TOP or TBP at room temperature. The cadmium precursor was prepared from cadmium oxide, trioctylphosphine oxide (TOPO) and hexadecylamine (HDA); the mixture was heated to 360ºC under flowing Ar to dissolve cadmium oxide. The solution was cooled to 270ºC and the selenium stock solution (Se/TOP or Se/TBP) was injected; nanocrystals grew to the desired size at 250ºC. The CdSe//TOPO/(TOP or TBP) nanoparticles were purified according to ref. [2]. Using this procedure, pure CdSe//TOPO/TOP or CdSe//TOPO/TBA QDs were obtained in the range of 2.4 – 4.3 nm.

##### *Preparation of ODE/OA-coated CdSe nanocrystals*

Hydrophobic ligands-coated CdSe nanocrystals were prepared from cadmium oxide and elementary selenium using reference procedure [3]. Briefly, the selenium precursor was prepared by combining elementary selenium and TOP at room temperature. The cadmium precursor was prepared from cadmium oxide, ODE, and oleic acid at 250 °C. Prepared solutions were mixed at different temperatures for the appropriate time period to obtain the desired size of the CdSe nanocrystal. The resulting CdSe//ODE/OA nanocrystals were purified similarly as in procedure above [2].

**References used in this section**

1. Peng ZA, Peng X (2001) Formation of High-Quality CdTe, CdSe, and CdS Nanocrystals Using CdO as Precursor. J Am Chem Soc 123:183-184
2. Yu WW, Qu L, Guo W, Peng X (2003) Experimental determination of the extinction coefficient of CdTe, CdSe, and CdS nanocrystals. Chem Mater 15:2854-2860
3. Asokan S, Krueger KM, Alkhawaldeh A, Carreon AR, Mu Z, Colvin VL, Mantzaris NV, Wong MS (2005) The use of heat transfer fluids in the synthesis of high-quality CdSe quantum dots, core/shell quantum dots, and quantum rods. Nanotechnology 16:2000-2011

**Biphase transfer method – graphical interpretation**

**
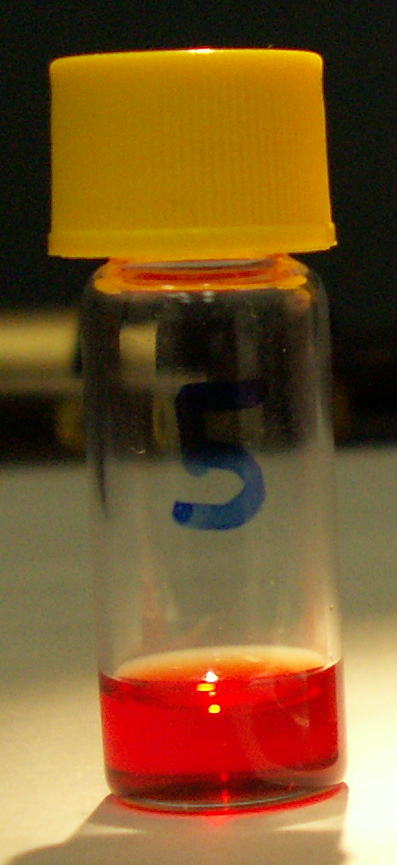

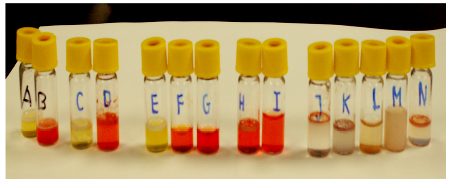
**

CdSe NCs dispersed in organic solvents (hexane, chloroform)

Aqueous solution of surfactant was added to form a biphase system

Evaporation of organic solvent

Surfactant molecules cover NCs to form aqueous soluble NC/surfactant

Fig. S1. Graphical illustration of the biphase transfer method. Case: coating of CdSe NCs with surfactants.

**Scale: PL per 0.1 absorbance unit (PL/0.1AU)**

In the present work the scale PL/0.1AU was adopted as a tool for analyzing surface phenomena for CdSe nanocrystals. The tool is based on the 0.1 absorbance unit (AU) because the concentration of semiconductor nanocrystals in solution should be as low as possible to avoid PL self-quenching and due to sort of experimental limitations that leads to low NCs dispersion, e.g. coating with non-ionic surfactants. In such situation 0.1 AU scale reflects absorbance level encountered experimentally. Note, that the PL/0.1AU tool was already applied in order to discuss impact of a surface ligand on PL of CdSe NC [ref. **I** below].

Construction of the PL/0.1AU tool is based on two relations. First one is normalized concentration (c_NC_ (mol l^-1^) per 0.1 AU) over CdSe NCs sizes, based on two equations from ref. [**II**]; namely eq. 14 (relation between λ_max_ of *first exciton absorption band* and NC size) and eq. 15 (relation between λ_max_ of *first exciton absorption band* and *molar extinction coefficient* (*ε*). Note, that ref. [**II**] re-examines discussion on this issue presented in ref. [**III**].

Fig. S2. Normalized CdSe NCs concentration per 0.1 absorbance unit (0.1AU/ε). The factor (0.1AU/ε) shows that depending on CdSe NCs size, the level of 0.1 absorbance requires different NCs concentration, e.g. ~ 6.5 x 10^-7^ mol l^-1^ for CdSe size 2 nm or ~ 2.3 x 10^-7^ mol l^-1^ for CdSe size 5 nm. Inset shows typical spectrum of CdSe NCs with data (NC size, ε and concentration) extracted from *first exciton absorption* band λ_max_ = 574 nm, based on eq. 14 and 15 from work [II]. Term E_g_ denotes the bandgap energy (eV) for CdSe NC.

Based on normalized NCs concentration over NCs sizes (Fig. S2), the PL/0.1 AU tool, thus PL counts pre normalized concentration (over NCs sizes), was established (Fig. S3). Although report [**IV**] shows that PL yield may not be constant over wide range of NCs sizes, the theoretical analysis [ref. **V**] along with discussed experimental examples shows that band gap (difference between λ_max_ (Abs) and λ_em_ (PL)) can be equal for wide range of NCs sizes. In such situation, PL dependence should be equally shifted over AU dependence via the normalized NCs concentration (Fig. S3). However, the intrinsic feature for NCs is the presence of surface defects that diminishes PL efficiency. Such feature is seen for CdSe NCs size < (2.2 – 2.5 nm) due to the large surface to volume ratio and because of this, the higher content of surface atoms (and importance of surface phenomena). Dashes red line (Fig. S3) reflects this situation. Note, this shape is in agreement with, e.g. work [ref. **VI**], where for CdTe NCs maximal level of PL was observed for NC size 3 nm.

Arbitrary units

**0.1AU/ε**; **PL/0.1AU**

Fig. S3. Construction of PL/0.1AU tool. Blue line: normalized concentration per 0.1 AU, over NCs sizes (0.1AU/ε). Black line: PL/0.1AU, thus PL counts = f(normalized NCs concentration), and dashed red line: PL/0.1AU for real CdSe NCs with surface defects at CdSe size < 2.2 nm.

The PL/0.1 AU tool was applied in the present work to visualize efficiency in NC surface coating, as well as at tool enable compare surface effects that impact PL over wide range of NCs sizes.

**References used in this section:**

[**I**] K. Nose, H. Fujita, T. Omata,, S. O.-Y.-Matsuo, H. Nakamura, H. Maeda, Chemical role of amines in the colloidal synthesis of CdSe quantum dots and their luminescence properties, J. Lumin. 126 (2007) 21–26.

[**II**] J. Jasieniak, L. Smith, J. van Embden, P. Mulvaney, Marco Califano, Re-examination of the Size-Dependent Absorption Properties of CdSe Quantum Dots, J. Phys. Chem. C, 113 (2009) 19468–19474.

[**III**] Yu WW, Qu L, Guo W, Peng X Experimental determination of the extinction coefficient of CdTe, CdSe, and CdS, nanocrystals. Chem. Mater. 15 (2003) 2854–2860.

[**IV**] L. Qu, X. Peng, Control of Photoluminescence Properties of CdSe Nanocrystals in Growth**,** J. Am. Chem. Soc. 124 (2002) 2049-2055.

[**V**] R. W. Meulenberg, J. R.I. Lee, A. Wolcott, J. Z. Zhang, L. J. Terminello, T. van Buuren, Determination of the Exciton Binding Energy in CdSe Quantum Dots, ACS Nano, 3 (2009) 325–330.

[**VI**] Y.-H. Zhang, H.-S. Zhang, M. Ma, X.-F. Guo, H. Wang, The influence of ligands on the preparation and optical properties of water-soluble CdTe quantum dots, Appl. Surf. Sci. 255 (2009) 4747–4753.

aggregation

Fig. S4. Dispersion of CdSe NCs (2.6 nm) in aqueous solution of sodium oleate (OA). The plateau in UV-vis absorbance (> 100 mM OA) denotes optimal conditions for NCs coating with the surfactant that yield in highest dispersion of CdSe NCs in OA surfactant. In the same range an electronic interaction ligand/surface of CdSe reached optimal level (PL = const.) at > 100 mM OA. The yellow box denotes the OA range where CdSe NCs aggregation occurs.

## Molecular modeling: interaction between CdSe nanocrystal and a ligand

In the present work DFT tool, previously examined in the work [Oszwałdowski et al. *Microchim. Acta* 176 (2012) 345], was applied in order to establish the binding between CdSe crystal and ligand in terms of binding energy. Calculation was based on C_6_ chain (ligand) that allows reduce the computation time, whereas preserving the most important interactions between CdSe crystal and ligand. Three most important crystal facets were considered.

Table S1: Binding energy for selected ligands vs. appropriate facet of CdSe crystal.

| ↓molecule facet→ | [110] | [001] | [00-1] |
| --- | --- | --- | --- |
| **anionic surfactants** |  |  |  |
| CH_3_-(CH_2_)_5_-SO_4_^-^ | > 0 | -38.25 | > 0 |
| CH_3_-(CH_2_)_5_-COO^-^ | -49.44 | -63.83 | > 0 |
| CH_3_CH_2_CH=CH(CH_2_)_2_COO^-^ | -49.57 | -63.43 | > 0 |
| S^-^-(CH_2_)_6_-COO^-^ | -52.10 | -101.68 | > 0 |
| Br-(CH_2_)_6_-COO^-^ | -50.80 | -31.34 | -44.29 |
| **cationic surfactants** |  |  |  |
| CH_3_-(CH_2_)_5_-NH_3_^+^ | -119.81 | -71.40 | -127.62 |
| CH_3_-(CH_2_)_2_-N^+^C_5_H_5_ | -103.64 | -80.80 | -103.54 |
| **non-ionic surfactants** |  |  |  |
| CH_3_(OCH_2_CH_2_)_3_OCH_3_ | -75.24 | -51.29 | -30.36 |
| H(OCH_2_CH_2_)_3_OH | -52.46 | -64.69 | -19.22 |
| **hydrocarbon chain** |  |  |  |
| CH_3_-(CH_2_)_4_-CH_3_ | -37.09 | -23.77 | -10.00 |
| CH_3_CH_2_-CH=CH-CH_2_CH_3_ | -33.96 | -32.35 | -9.52 |
| CH_2_=CH-CH_2_CH_2_-CH=CH_2_ | -104.96 | -105.84 | -136.88 |
| **typical ligands for CdSe** |  |  |  |
| DHLA | -198.70 | -148.81 | -125.72 |
| MPA | -60.73 | -106.90 | -115.8 |
| PC3 | -49.41 | -13.35 | -20.87 |
| OPC3 | -38.09 | -14.86 | -2.8 |
| py | -44.44 | -33.23 | -5.68 |

Binding energy (kcal mol^-1^) = energy (surface-ligand) – energy [(surface) + (ligand)]. (DFT) Dmol^3^ module. Functional: PWC/LDA (Pedrew-Wang (1992) LDA), convergence tolerance – energy 2 x 10^-5^ Ha, SCF tolerance 1 x 10^-5^, core treatment – all electrons, basic set DND, orbital cutoff – medium. MPA = 3-mercaptopropionic acid, DHLA = dihydrolipoic acid,. py = pyridine, O=P(R)_3_ = trialkylphosphine oxide (alkyl = C_1_); P(R)_3_ = trialkylphosphine, (alkyl = C_1_).

# CdSe – ligand CdSe – pyridine CdSe – ligand E > 30 - 40 kcal/mol E ~ 30 - 40 kcal/mol E < 40 kcal/mol

**binding** **threshold** **non-binding**


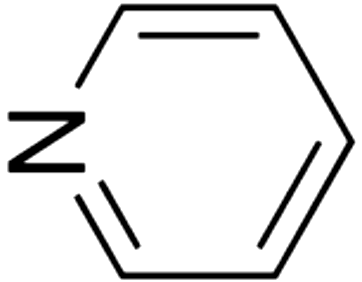


original surface ligand (e.g. TOPO, TOP)

surfactant

Fig. S5 Graphical interpretation of coating of CdSe NCs, passivated by hydrophobic ligands, with binding and non-binding ligands vs. threshold ligand, taking into account data from theoretical model (Table S1, ESM).

**AU** **PL** (counts)

**Wavelength (nm)**

TOPO/TBP

TOPO/TOP

**a**

**b**

Fig. S6. Comparison of coating of CdSe NCs with CTAB. CdSe NCs were obtained from TOPO/TBP (frame **a**) or TOPO/TOP synthesis (frame **b**). Samples of NCs in hexane (A) were dispersed in 100 mM CTAB (B). Right column UV-vis spectra, left column PL spectra, respectively. Note, that R-NR_3_^+^ cation, electro-accepting ligand, quenches PL of CdSe NCs.

The experiment relies on transfer NCs from hexane to aqueous phase with CTAB assistance (Fig. S6). Two types of CdSe NCs were considered, those containing TOPO/TBP and TOPO/TOP surface ligands. It can be concluded from Fig. S6 that CTAB is able to coat and transfer TOPO/TBP passivated NCs to aqueous solution, whereas NCs passivated with TOPO/TOP were less effective.

The main difference between these two NCs is TBP vs. TOP surface ligands. Based on the experimental results binding of the ligand to CdSe should follow the order: TBP < CTAB < TOP (in terms of binding energy and kinetics of ligand exchange). Uniquely, CTAB is able to replace TBP on the NC surface and unable to do so with TOP. It is important to note that these ligands (TBP, TOP and CTAB) are bound to the Se atom of CdSe nanocrystals, thus such comparison is reasonable. Therefore, from these results it can be stated that for TOPO/TBP NCs, CTAB behaves as a binding ligand, whereas for TOPO/TOP CTAB behaves as non-binding ligand, able to coat the TOPO/TOP surface through van der Waals interactions, to provide water solubility.

Fig. S7. Coating CdSe NCs with surfactants (~coating efficacy with surfactants). Nanocrystals according to synthesis: CdSe (TOPO/TBP) upper; CdSe (ODE/OA) below. Surfactants: A-E common for both examples; A – CdSe in chloroform, B – sodium oleate 100 mM; C – AOT, 100 mM; D – N-101 (5%); E – CTAB 100 mM. For the NC upper: F, sodium laurate 100 mM; G – TX-100 (5%); H – SDS 100 mM. For the NCs below: F - non-ionic surfactants, 5% (TX-100, TX-114, TX-100R, TX-405R, Brij (35, 56, 58, 700), Tween (20, 60), Igepal DM-970) and ionic, 100 mM (SDS, CP). Examples (A vs. A`) upper mean that the difference in the concentration of CdSe does not affect position of wavelength (λ_max_) PL.

All experiments dealing with NCs transfer from organic to aqueous phase were done in the same way. To the 0.3 ml of surfactant solution 50 μl of CdSe NCs solution in hexane (c_NC_ ~ 10^-5^ M) was added. The mixture was stirred overnight in order to evaporate the organic solvent that enable NCs coating with a surfactant. After this, samples were centrifuged in order to remove aggregates and clear samples were measured by optical techniques. Note, that well defined baseline in UV-vis spectra in the range above λ_max_ is a sign that the sample is aggregate–free.

**Etching of CdSe NCs by N-101 non-ionic surfactant**

Surfactant assisted dispersion of CdSe NCs in aqueous solution can lead to etching of the core of CdSe NC. The outstanding example is N-101 surfactant, where etching of core of CdSe NC was frequently observed. This irrespective of the way of NC synthesis or conditions for biphase NC transfer (organic → aq. surfactant; e.g. kind of organic solvent: chloroform vs. hexane). Fig. S8 shows the comparison of two structurally similar non-ionic surfactants (TX-100 vs. N-101). The blue shift in either UV-vis and PL spectra, related with NC core etching, was seen for N-101 using the same dispersion conditions for both surfactants. This regardless to CdSe NCs core sizes (inset frame a, Fig. S8), expressed in terms of λ_max_ of *first exciton band* in UV-vis range.

n = 9 -10

## TX-100 N-101

**CdSe → CdSe/N-101**

578 nm 545 nm

578 nm 522 nm

594 nm 567 nm

516 nm 473 nm

570 nm 539 nm

**a**

**b**

**Wavelength (nm)**

Fig. S8. Etching of core of CdSe NC due to dispersion of CdSe NCs in the non-ionic surfactants. Case: TX-100 vs. N-101. Inset frame **a** shows examples of etching using N-101 surfactant. Samples: A, CdSe NC chloroform solution and the sample dispersed in, B, TX-100 and C, N-101. Frames: **a**, UV-vis spectra and **b**, PL spectra.

The phenomenon observed by optical spectra was supported by TEM analysis (Fig. S9), where it was found that irrespective to CdSe NC core size, dispersion of CdSe NC using N-101 leads to core etching ca. 0.5 nm, compared with initial core diameter, i.e. CdSe NCs dispersed in organic solvent.


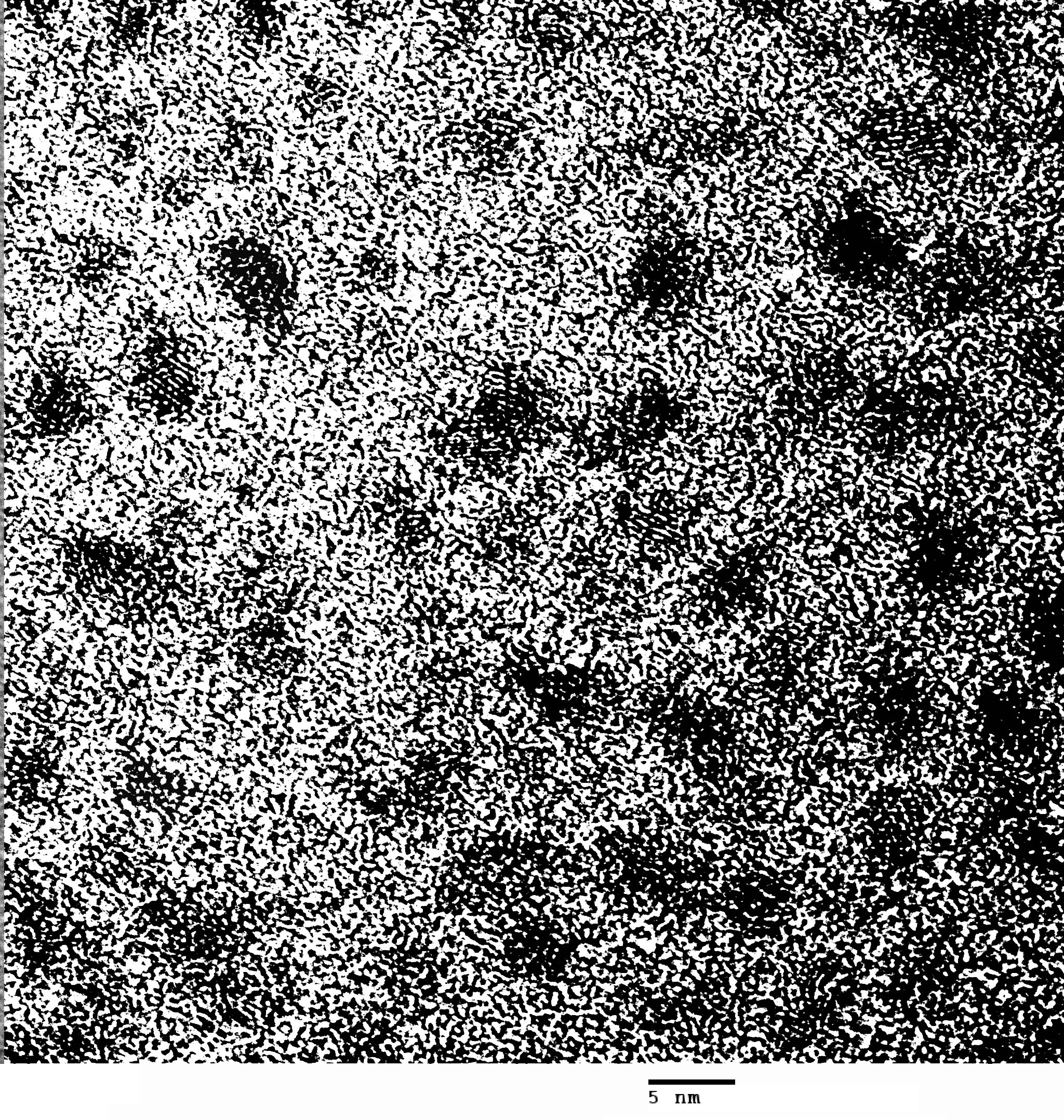

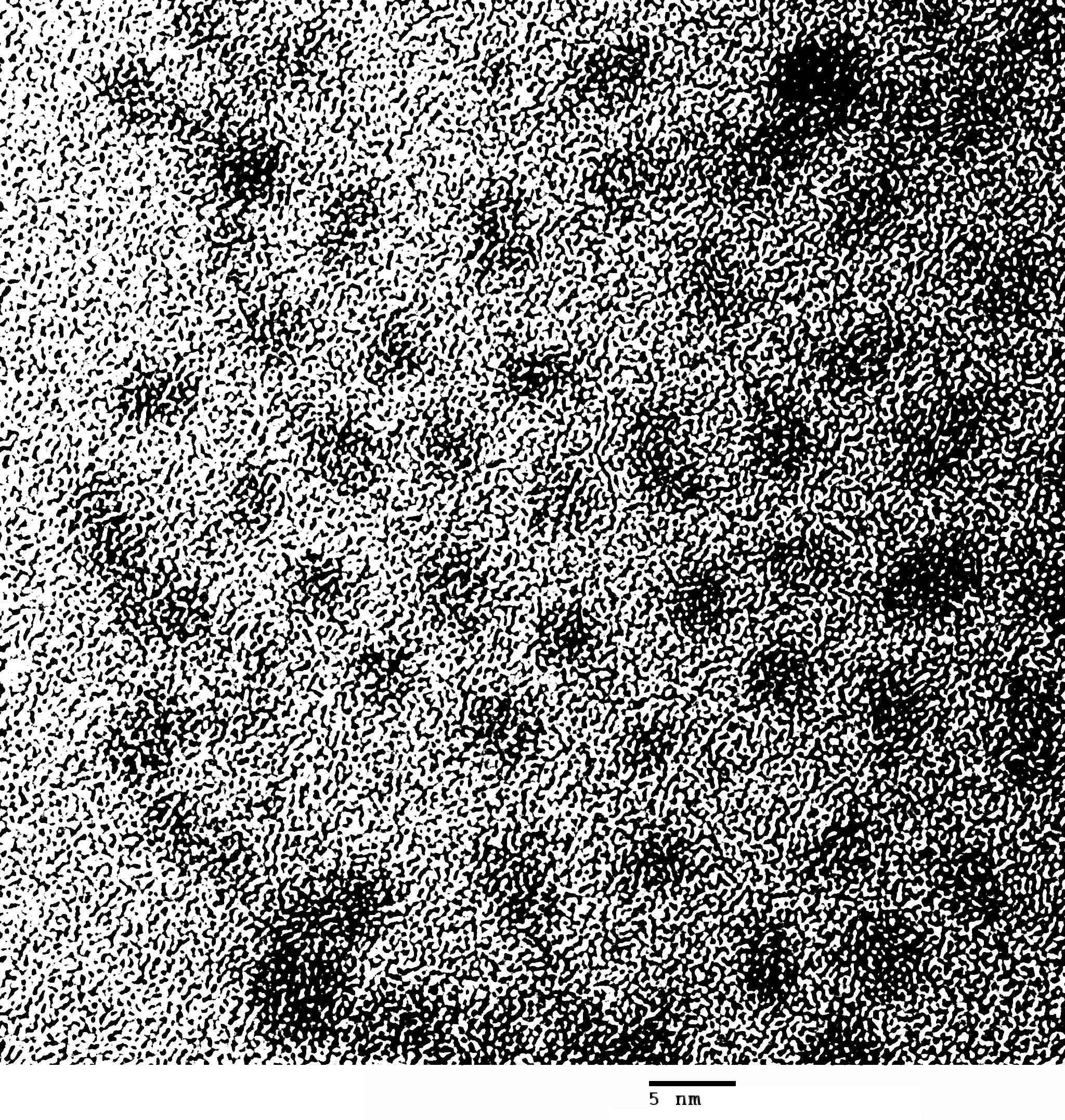


TEM/ JEOL JEM-2100

CdSe/TOP CdSe/TOP/N-101

TEM/ Hitachi H7000

CdSe//TOP CdSe/TOP/N-101

Fig. S9. TEM analysis of CdSe//TOPO/TOP NCs dispersed in hexane (white bars) and these NCs dispersed in aqueous solution of N-101 surfactant (gray bars).

In our opinion action of N-101 can be regarded as useful tool to distinguish the quality of CdSe crystal or its surface passivation.

Fig. S10. PL efficiency in terms of PL/0.1 AU for CdSe NCs (TOPO/TBP or /TOP) and these CdSe NCs modified by non-ionic surfactants. Brown (leftmost) bars show PL/0.1 AU for CdSe//TOPO/TBP NCs dispersed in hexane. Narrowed bars show PL/0.1 AU for these NCs coated with the most efficient surfactants, according to Fig. 2: (yellow) DM-970; (red) Brij 78; (green) Brij 35 and (blue) TX-405R. Solid line shows the average PL/0.1 AU obtained for six samples of CdSe NCs, obtained from independent syntheses. NCs with lowest size (2.4 nm) was omitted due to either low dispersion in aqueous solution of non-ionic surfactants and the lowest extinction coefficient for small CdSe NCs. Both preclude reliable spectra for NCs modified by non-ionic surfactants in this size range.

In the figure, the solid black line represents PL/0.1 AU obtained from measuring hexane solution of either TOPO/TBP or TOPO/TOP capped CdSe NCs (six samples per each NC size). As established in the discussion of Figure 2, it can be concluded that non-ionic surfactants retain high PL for CdSe NCs in aqueous medium. Figure S10 confirms that PL efficiency, using these surfactants, is comparable to PL efficiency for NCs, obtained from the optimized synthesis. Therefore, non-ionic surfactants can be regarded as efficient capping ligands in aqueous media, in terms of electronic interaction between surface ligands and the NC surface. This is believed to be related to either the quality of the original NC surface or its degree of passivation. An interesting example is shown in Figure S10 for 3.4 nm CdSe NCs coated with non-ionic surfactants. The initial PL of CdSe nanocrystal is lower than expected, but upon coating with a non-ionic surfactant, PL efficiency is restored. This indicate that surface defects, which cause non-radiative carrier recombinations, were eliminated by coating with these surfactants, despite the change in NCs environment from organic to aqueous.

Fig. S11. Optical effects (UV-vis, PL) due to coating CdSe NCs with selected non-ionic surfactants. Samples: A, CdSe//TOPO/TBP dispersed in hexane and this sample dispersed in B, Brij 700; C, Brij 56 and D, Igepal DM-970. Note, that a red shift in λ_em_ PL vs. fixed λ_max_ was observed for surfactant coated CdSe NCs vs. starting CdSe//TOPO/TBP. The red shift is related with PL efficiency (see inset). This issue is discussed in Section of the body text: *Bathochromic shift in λ_max_ of photoluminescence (PL) for semiconductor CdSe nanocrystals with the same energy gap* (*E_g_*).

## AU

## PL

Δλ = 11 nm

A

B

A

A`

B

CdSe 2.5 nm

CdSe 2.9 nm

**a**

**b**

**Wavelength (nm)**

Fig. S12. Spectral difference (λ_em_ – λ_Abs_ = Δλ) for CdSe nanocrystals obtained from: (A) TOPO/TBP and (B) TOPO/TOP synthesis. Nanocrystal core dimension: frame (**a**) 2.5 nm, (**b**) 2.9 nm. Frame **b** shows example of independence of Δλ on CdSe nanocrystal concentration in the range up to 2.3 x 10^−6^ M (CdSe//TOPO/TBP NCs). Nanocrystals concentration (M): frame (**a**) A, 3.2 x 10^−6^; B, 1.3 x 10^−6^; frame (**b**) A, 2.3 x 10^−6^; A`, 1.0 x 10^−6^; B, 0.9 x 10^−6^. NCs dispersion in chloroform.

**Bathochromic shift in λ_max_ of photoluminescence (PL) for semiconductor CdSe nanocrystals with the same energy gap (*E_g_*)**

Considering literature references it can be concluded that λ_em_ PL shift at constant λ_max_ (Abs) is originated from unrelated from each other phenomena, e.g. solvent permittivity, crystal shape or due to energy transfer, that a proper classification of the effect is not feasible. Generally, the literature survey, despite vast literature positions dealing with semiconductor nanocrystals, confirms only a few references that notice the feature. At least four options dealing with a red shift in λ_em_ PL at constant λ_max_ (Abs) should be considered:

**I.** Works [1,2] discuss relation between λ_em_ (PL) and shape of CdSe or CdTe NCs. For the same absorption *λ_max_*, despite difference in nanocrystals shape (dots vs. rods), the bathochromic shift in λ_em_ PL for rods vs. dots was observed.

**II.** Works [3-5] discuss shift in λ_em_, due to dispersion of CdSe/ZnS NCs, that involves only interaction between native ligand and dispersant with the conclusion that a surface phenomena are decisive for Δ λ_em_.

**III.** Works [6-8] discuss effect of nanocrystal aggregation or polymerization. In this instance the most profound effect is the energy transfer between particles about different sizes.

**IV.** Works [9-12] discuss change in λ_em_, due to exchange of surface ligands. Because of contradictory reports it is difficult to withdrawn here one general conclusion.

#### References

[1] W.W. Yu, Y.A.. Wang, X. Peng, Formation and Stability of Size-, Shape-, and Structure-Controlled CdTe Nanocrystals: Ligand Effects on Monomers and Nanocrystals, *Chem. Mater.* **2003,** *15,* 4300-4308

[2] X. Peng, L. Manna, W. Yang, J. Wickham, E. Scher, A. Kadavanich, A.P. Alivisatos, Shape control of CdSe nanocrystals, *Nature* **2000**, 404, 59-61

[3] J. Feng, S-Y. Ding, M.P. Tucker, M.E. Himmel, Y-H. Kim, S.B. Zhang, B.M. Keyes, G. Rumbles, Cyclodextrin driven hydrophobic/hydrophilic transformation of semiconductor nanoparticles, *Appl. Phys. Lett*. **2005**, 86, 033108-1 - 033108-3

[4] T. Jin, F. Fujii, E. Yamada, Y. Nodasaka, M. Kinjo, Control of the Optical Properties of Quantum Dots by Surface Coating with Calix[n]arene Carboxylic Acids, *J. Am. Chem. Soc*. **2006**, 128, 9288-9289

[5] H. Fan, E.W. Leve, Ch. Scullin, J. Gabaldon, D. Tallant, S. Bunge, T. Boyle, M.C. Wilson, C.J. Brinker, Surfactant-Assisted Synthesis of Water-Soluble and Biocompatible Semiconductor Quantum Dot Micelles, *Nano Lett*. **2005,** 5, 645-648

[6] M. Noh, T. Kim, H. Lee, Ch-K. Kim, S-W. Joo, K. Lee, Fluorescence quenching caused by aggregation of water-soluble CdSe quantum dots, *Colloids Surf., A* **2010**, 359, 39–44

[7] R. Koole, P. Liljeroth, C de Mello Donega´, D. Vanmaekelbergh, A. Meijerink, Electronic Coupling and Exciton Energy Transfer in CdTe Quantum-Dot Molecules, *J. Am. Chem. Soc*. **2006**, 128, 10436-10441

[8] X. Xu, S. Stȍttinger, G. Battagliarin, G. Hinze, E. Mugnaioli, Ch. Li, K. Müllen, T. Basch, Assembly and Separation of Semiconductor Quantum Dot Dimers and Trimers, *J. Am. Chem. Soc*. **2011**, 133, 18062–18065

[9] W.R. Algar, U.J. Krull, Luminescence and Stability of Aqueous Thioalkyl Acid Capped CdSe/ZnS Quantum Dots Correlated to Ligand Ionization, *ChemPhysChem* **2007**, 8, 561 – 568

[10] C. Bullen and P. Mulvaney, The Effects of Chemisorption on the Luminescence of CdSe Quantum Dots, *Langmuir* **2006,** 22*,* 3007-3013

[11] P.K. Sharma, R.K. Dutta, Ch.H. Liu, R. Pandey, A.C. Pandey, Surfactant mediated optical properties of cytosine capped CdSe quantum dots, *Material. Lett*. **2010**, 64, 1183–1186

[12] Y. Liang, J. E. Thorne, B. A. Parkinson, Controlling the Electronic Coupling between CdSe Quantum Dots and Thiol Capping Ligands via pH and Ligand Selection, Langmuir **2012**, 28, 11072−11077

TOPO

DOSS

OA

SDS

SS

CTAB

Tween

Fig. S13. CdSe//TOPO/TBP NCs dispersed in various surfactants under UV light. Samples from left: CdSe NCs dispersed in hexane (size 2.6 nm: λ_max_ 516 nm, λ_em_ 532 nm) and these NCs dispersed in AOT, OA SDS, sodium stearate (SS), CTAB and Tween 60.

**Wavelength (nm)**

Fig. S14. Effect of coating of CdSe//TOPO/TBP NCs with OA surfactant in terms of the red shift in λ_em_, as well as PL/0.1AU factor. Left column, UV-vis spectra and right column, photoluminescence spectra, respectively. Sizes of CdSe (nm): **a**, 2.6; **b**, 3.1 and **c**, 5.1. Insets frames **a-c** shows PL/0.1AU for each situation. Frame **b** shows that either the position of λ_em_ PL or PL/0.1AU is independent on NCs concentration (samples: A, B). Frame **c** shows example of the blue shift in λ_em_, which is related with etching of NC core, irrespective of the surfactant applied for coating (OA, LA). In this case a decrease in PL efficiency can be 10-fold.

a

b

c

Fig. S15. UV-vis and PL spectral features for upper phase of cloud-point extraction system. Sample applied: 2.9 nm CdSe NCs dispersed in TX-114 solution. TX-114 concentration applied: A, 2.5%, B, 5% and C, 7% (w:w) (see Fig. 6 trace **a**). The UV-Vis spectra were similar for all these samples in terms of absorbance and λ_max_. The initial CdSe NCs solution (2.9 nm CdSe dissolved in hexane) applied for samples preparation: λ_max_ = 543 nm (UV-Vis), λ_max_ = 559 nm (PL) and PL/0.1 AU = 21022.

**CdSe**

Fig. S16. Graphical interpretation of the attachment of a non-ionic surfactant to surface of smaller vs. bigger CdSe nanocrystal that result in the nanocrystal presence in upper vs. bottom phase, respectively, due to the cloud-point extraction. Dashed boxes denote the most significant interactions between a surfactant and a surface of the nanocrystal in both cases.

**CdSe**
